# Linking Kawasaki Disease to Mental Health: A Nationwide Study on Long-Term Neurological Risks

**DOI:** 10.3390/medicina61040604

**Published:** 2025-03-26

**Authors:** Ji-Ho Lee, Taewoo Shin, Jung-Min Park, Jae-Hee Seol

**Affiliations:** 1 Department of Internal Medicine, Yonsei University Wonju College of Medicine, Wonju 26426, Republic of Korea; airwayleejh@yonsei.ac.kr; 2 Department of Pediatrics, Yonsei University Wonju College of Medicine, Wonju 26426, Republic of Korea; staeoo@yonsei.ac.kr; 3 Department of Pediatrics, National Health Insurance Service Ilsan Hospital, Goyang 10444, Republic of Korea; 4 Department of Pediatrics, Yonsei University College of Medicine, Seoul 03722, Republic of Korea

**Keywords:** Kawasaki disease, mental health, neuropsychiatric disorder, neurodevelopmental disorder, nationwide study

## Abstract

*Background and Objectives*: Kawasaki disease (KD) is a childhood systematic vasculitis. Emerging evidence suggests a link between KD and long-term neurological implications. This study examines the association between KD and subsequent neuropsychiatric and neurodevelopmental disorders using national health data from South Korea. *Materials and Methods*: Using the National Health Information Database, we identified KD patients diagnosed between 2002 and 2021 and selected those born between 2008 and 2015. Propensity score matching with a 1:4 ratio was applied to create a control group. The incidence of neuropsychiatric and neurodevelopmental disorders from 2017 to 2021 was analyzed using Cox proportional hazard models, adjusting for age, sex, and urbanicity. *Results*: This study included 41,806 KD subjects and 163,829 matched controls. KD was associated with an increased risk of certain neuropsychiatric disorders: anxiety disorder (HR: 1.124, 1.047–1.207), sleep-related disorder (HR: 1.257, 1.094–1.444), movement disorder (HR: 1.227, 1.030–1.461), and any neuropsychiatric disorder (HR: 1.102, 1.053–1.153). For neurodevelopmental disorders, KD patients showed a lower incidence of intellectual disability (HR: 0.747, 0.641–0.871) but an increased risk of tic disorder (HR: 1.148, 1.020–1.292). Male gender and urban residency were associated with higher incidence rates for certain conditions. *Conclusions*: This study demonstrates that KD patients show increased risks for anxiety, sleep-related disorder, movement disorder, and tic disorder, a reduced incidence of intellectual disability, and a higher risk of tic disorder. These findings highlight the need for long-term neurological monitoring in KD patients and provide insights into its potential neurodevelopmental impact.

## 1. Introduction

Kawasaki disease (KD) is an acute systemic vasculitis primarily affecting children, with cardiac complications being the major focus of its long-term prognosis [1]. As KD predominantly involves medium-sized blood vessels, it can lead to serious cardiovascular complications, including coronary artery aneurysms, myocarditis, and valvulitis. In addition to cardiac involvement, KD is associated with various non-cardiac complications, including gastrointestinal manifestations, such as abdominal pain and intestinal ischemia, ophthalmologic complications, such as uveitis, and musculoskeletal involvement, such as acute polyarthritis [2,3,4]. KD is also associated with various neurological manifestations, such as seizures, hearing loss, facial nerve palsy, and paralysis, which suggest the potential involvement of the central nervous system [5,6]. Previous studies have explored KD-related neurological complications using diverse approaches, such as cerebrospinal fluid (CSF) pro-inflammatory cytokine analyses and single-photon emission computed tomography (SPECT) imaging, that revealed acute-phase hypoperfusion in KD patients [7,8,9]. Additionally, an electroencephalography (EEG) study found that KD patients exhibited a lower alpha peak amplitude ratio, indicating potential long-term functional impairment [10]. These findings suggest that underlying neuroinflammatory mechanisms may contribute to the risk of neurological complications in patients with KD.

Emerging evidence suggests that immune dysregulation and chronic inflammation play a critical role in the pathogenesis of psychiatric and neurodevelopmental disorders [11,12,13,14]. Studies on autoimmune diseases like systemic lupus erythematosus, rheumatoid arthritis, and ankylosing spondylitis suggest a strong association between chronic inflammatory conditions and psychiatric disorders [15,16,17]. Atopic dermatitis and asthma have also been linked to mental health conditions [18]. Given KD’s inflammatory nature, it is plausible to hypothesize that KD may similarly predispose patients to neuropsychiatric and neurodevelopmental disorders, thereby influencing its long-term mental health outcomes. This highlights the importance of early intervention and long-term monitoring in KD survivors.

Previous studies, including nationwide cohort studies from Taiwan and Canada, have attempted to explore these associations [19,20,21,22,23,24,25,26]. While these studies provided valuable insights, they were limited by small sample sizes, reliance solely on diagnostic codes, and a narrow focus on specific disorders. Additionally, their findings on the relationship between KD and neuropsychiatric disorders have been inconsistent. The multifactorial etiology of neuropsychiatric diseases—involving genetic predispositions, immune system interactions, environmental exposures, and social determinants—makes it challenging to establish a definitive causal link between KD and these disorders [27,28]. Moreover, as most of the existing studies were conducted in specific populations, their findings may lack generalizability to other regions and ethnic groups. Given that neurodevelopmental disorders have significant long-term consequences on quality of life, a more comprehensive investigation is necessary. Notably, no studies have yet examined this association in the Korean population. Therefore, this study aims to analyze data from the Korean National Health Insurance Service (NHIS) to comprehensively assess the association between KD and neuropsychiatric and neurodevelopmental disorders. By utilizing a comprehensive, large-scale, nationwide dataset and systematically comparing a wide range of these disorders, this study not only provides a clearer understanding of which conditions are associated with KD but also establishes an epidemiological foundation for exploring potential underlying mechanisms. The findings may contribute to future research on KD-related pathophysiology and inform long-term follow-up and management strategies.

## 2. Materials and Methods

### 2.1. Data Source

The South Korean healthcare system boasts a comprehensive public health database known as the National Health Information Database (NHID), overseen by the NHIS [29]. This extensive repository encompasses data on more than 50 million individuals, dating back to 2002, and includes information on healthcare utilization, health screenings, socio-demographic factors, and mortality rates. The NHID is composed of several interlinked databases, including eligibility, national health screening, healthcare utilization, long-term care insurance, and healthcare provider information. These databases offer detailed insights into insurance contributions, demographic data, health behaviors, medical treatments, prescription records, and healthcare facilities. To ensure privacy, the NHID employs deidentified join keys to connect these databases. This valuable resource has been instrumental for researchers investigating various diseases, health conditions, and risk factors and the impact of health policies. Access to the NHID is granted through a formal application process, which necessitates ethical approval and review by the NHIS committee.

### 2.2. Study Design and Population

**Figure 1:**
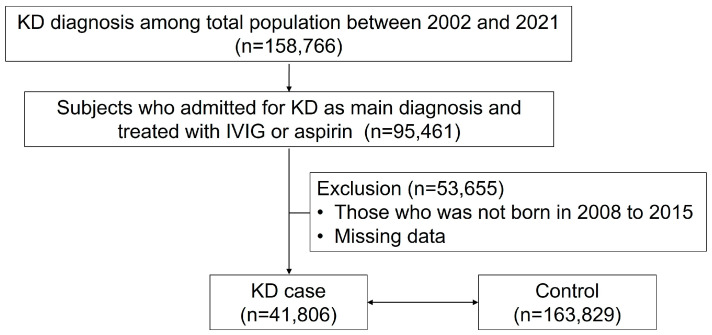
Patient selection flow.

This study identified individuals diagnosed with KD using the M303 diagnostic code between 2002 and 2021. The researchers focused on patients admitted with KD as the primary diagnosis who received intravenous immunoglobulin or aspirin treatment. The cohort was further refined to include only those born between 2008 and 2015. The cohort of children born between 2008 and 2015 was selected from the NHIS database, which was established in 2002. To minimize potential errors in ICD registration during the initial transition period, a five-year window was applied. Additionally, the cohort was limited to births up to 2015 to ensure a sufficient follow-up duration for the study outcome period (2017–2021). To establish a control group, researchers screened individuals without a history of KD and applied 1:4 propensity score matching to match them with the study group based on age, sex, and urbanicity (Figure 1). This method facilitated a balanced comparison between KD cases and controls, reducing potential confounding variables.

### 2.3. Study Outcomes

In our study, we categorized neurologic disorders into two main groups: neuropsychiatric disorders and neurodevelopmental disorders. The neuropsychiatric disorder category encompassed seven conditions: psychotic disorder, mood disorder, anxiety disorder, sleep-related disorder, cognitive disorder, movement disorder, and personality disorder. The neurodevelopmental disorder category included six conditions: intellectual disability, communication disorder, specific learning disorder, autism spectrum disorder, attention deficit hyperactivity disorder (ADHD), and tic disorder. We identified these disorders using their corresponding ICD-10 diagnostic codes, which are detailed in Table S1 [30,31]. Our observational period for assessing the incidence and prevalence of these neurologic disorders spanned from 2017 to 2021. We defined incident cases as newly diagnosed neurologic disorders within this observational period. Prevalent cases were considered the total number of subjects who received treatment for neurologic disorders during the same period.

### 2.4. Ethics

This study was conducted in accordance with the Declaration of Helsinki and was approved by the Institutional Review Board of Wonju Severance Christian Hospital (CR322347, approval date: 6 December 2022). Given the retrospective nature of this study utilizing anonymized claims data, the requirement for informed consent was waived.

### 2.5. Statistical Analysis

In our statistical analysis, we compared the baseline characteristics of the KD cases and controls using the Mann–Whitney U test for continuous variables and the chi-square test for categorical variables. We employed propensity score matching with a caliper width of 0.3 and the greedy matching method to identify KD cases and controls. To assess the incidence of neurologic disorders, we utilized a Cox proportional hazard model, expressing the results as hazard ratios (HRs) with 95% confidence intervals. For prevalence assessment, we applied a logistic regression model, presenting the outcomes as odds ratios (ORs) with 95% confidence intervals. We utilized both statistical models to thoroughly investigate the epidemiological association between the two diseases and to confirm the robustness of our results across different statistical methods. In our multivariate analysis of both HRs and ORs, we adjusted for all variables, including age, sex, and urbanicity. All statistical analyses were performed using SAS 9.4 (SAS Institute Inc., Cary, NC, USA). We considered a *p*-value less than 0.05 to be statistically significant.

## 3. Results

### 3.1. Demographics of Study Participants

In our study of KD in Korea, we initially identified 158,766 subjects with a KD diagnosis from 2002 to 2021, drawn from a population of approximately 50 million (Figure 1). To enhance the accuracy of our cohort, we narrowed our focus to 95,461 patients who were admitted with KD as the primary diagnosis and received KD-specific treatment. Our final KD cohort consisted of 41,806 subjects born between 2008 and 2015. Using a 1:4 ratio, we identified 163,829 matched controls.

Table 1 provides a comprehensive overview of the baseline characteristics for our study participants. The KD patients had a mean age of 2.63 ± 1.84 years, while the control group had a mean age of 2.64 ± 1.85 years (*p* = 0.119). In terms of gender distribution, females made up 42.20% of the KD group and 42.55% of the control group, with no significant difference between the two cohorts (*p* = 0.199). When examining the urban–rural split, we found that 67.11% of the KD group resided in urban areas, compared to 66.53% of the control group (*p* = 0.025).

### 3.2. Risk of Neuropsychiatric Disorders

**Figure 2:**
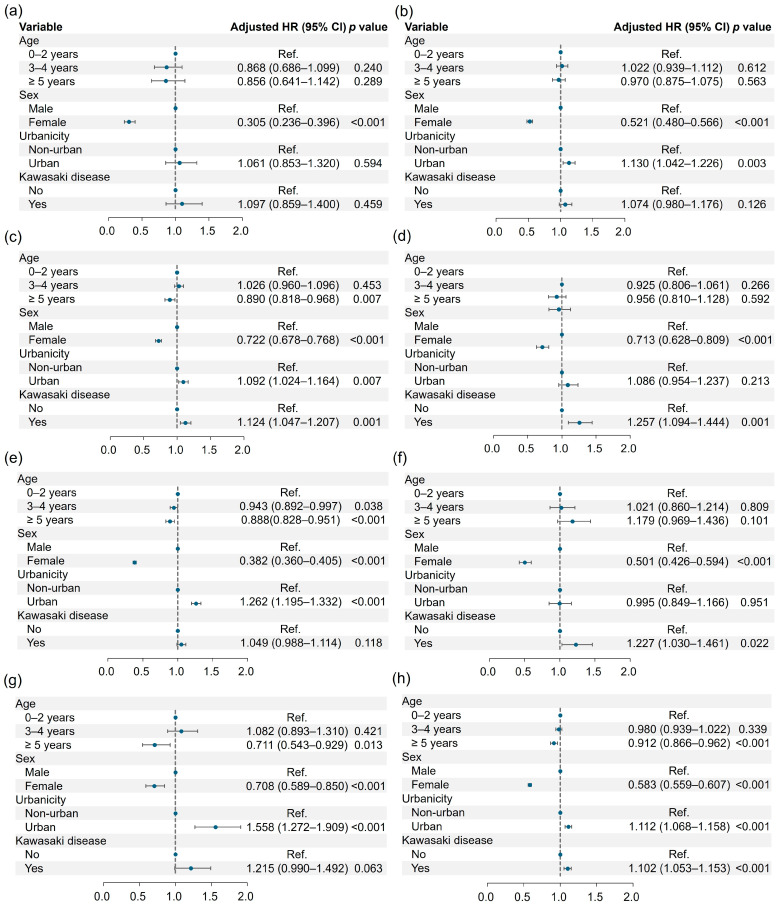
Hazard ratios of psychotic disorder (**a**), mood disorder (**b**), anxiety disorder (**c**), sleep-related disorder (**d**), cognitive disorder (**e**), movement disorder (**f**), personality disorder (**g**), and any neuropsychiatric disorder (**h**) according to the demographics and Kawasaki disease.

KD was associated with an increased risk of developing certain neuropsychiatric disorders. Specifically, children with KD had approximately a 12% higher risk of anxiety disorder (HR: 1.124, 1.047–1.207), a 26% higher risk of sleep-related disorder (HR: 1.257, 1.094–1.444), and a 23% higher risk of movement disorder (HR: 1.227, 1.030–1.461). Overall, KD was associated with roughly a 10% increased risk of developing any neuropsychiatric disorder (HR: 1.102, 1.053–1.153) (Figure 2). No statistically significant association was found between KD and the development of psychotic disorder, mood disorder, cognitive disorder, or personality disorder. The detailed incidence results and the association of neuropsychiatric disorders with KD are presented in Table 2. Gender had a notable impact, indicating that female correlated with lower incidences of all neuropsychiatric disorders. Urban residency was associated with higher incidence rates for mood disorder, anxiety disorder, cognitive disorder, and personality disorder.

Our analysis of prevalence yielded results consistent with those observed in the incidence study. KD patients exhibited approximately an 11% higher likelihood of having anxiety disorder (OR: 1.112, 1.037–1.192), a 33% higher likelihood of having sleep-related disorder (OR: 1.328, 1.163–1.518), and a 32% higher likelihood of having movement disorder (OR: 1.324, 1.120–1.565). Overall, KD patients demonstrated roughly a 10% increased likelihood of having any neuropsychiatric disorder (OR: 1.104, 1.057–1.153). Detailed prevalence results are presented in Table S2.

In summary, KD was associated with an increased risk and prevalence of certain neuropsychiatric disorders, particularly anxiety, sleep-related, and movement disorders. Gender and urban residency also influenced the incidence of specific neuropsychiatric conditions.

### 3.3. Risk of Neurodevelopmental Disorders

**Figure 3:**
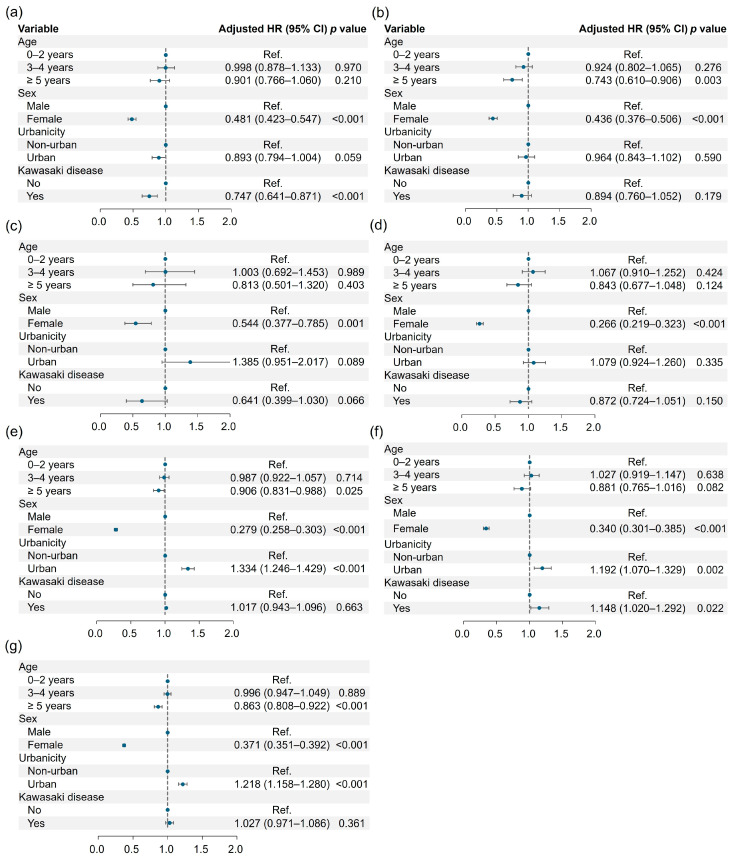
Hazard ratios of intellectual disability (**a**), communication disorder (**b**), specific learning disorder (**c**), autism spectrum disorder (**d**), ADHD (**e**), tic disorder (**f**), and any neurodevelopmental disorder (**g**) according to the demographics and Kawasaki disease.

We observed that individuals with KD had approximately a 25% lower risk of developing intellectual disability compared to those without KD (HR: 0.747, 0.641–0.871). Conversely, KD was associated with a 15% higher risk of developing tic disorder (HR: 1.148, 1.020–1.292) (Figure 3). However, KD was not associated with a risk of communication disorder, specific learning disorder, autism spectrum disorder, ADHD, or any neurodevelopmental disorder. The detailed incident results and the association of neurodevelopmental disorders with Kawasaki disease are presented in Table 3. Female gender was consistently correlated with lower incidence rates across all the neurodevelopmental disorders examined. In contrast, urban residency was associated with higher incidence rates for ADHD, tic disorder, and any neurodevelopmental disorder in general.

The prevalence analysis yielded results that aligned with our incidence findings. KD patients were approximately 23% less likely to have an intellectual disability compared to the controls (OR: 0.771, 0.674–0.883). However, KD patients exhibited a 17% higher risk of tic disorder (OR: 1.172, 1.046–1.312). The detailed prevalence results are presented in Table S3.

In summary, Kawasaki disease was associated with a lower risk of intellectual disability but a higher risk of tic disorder. Gender and urban residency also emerged as influential factors in the incidence and prevalence of these conditions.

## 4. Discussion

The findings of our study provide important insights into the association between KD and neurological disorders in children. Our results demonstrate significant associations between KD and specific neuropsychiatric and neurodevelopmental disorders, highlighting the potential long-term neurological implications of this acute systemic vasculitis. As categorized in our study, neuropsychiatric disorders include mood, cognitive, and behavioral conditions, whereas neurodevelopmental disorders involve impairments in early brain developments. Compared to previous cohort studies from Taiwan and Canada, our study utilized a large-scale, nationwide dataset and applied stringent diagnostic criteria by including only KD patients who received IVIG or aspirin therapy. This approach substantially enhances the accuracy of KD diagnosis, thereby minimizing potential confounding from misclassified cases. Furthermore, by conducting a comprehensive evaluation of both neuropsychiatric and neurodevelopmental conditions, our study offers a more comprehensive understanding of the long-term neurological impact of KD across various disorders, rather than limiting the analysis to a few selected conditions.

Our analysis revealed an increased risk of anxiety disorder, sleep-related disorder, movement disorder, and overall neuropsychiatric disorders in children with a history of KD. These findings align with the growing body of evidence suggesting a link between inflammatory conditions and psychiatric disorders [5]. The association between KD and anxiety disorder is particularly noteworthy, as it may indicate a lasting impact of the acute inflammatory process on the emotional regulation and stress response systems. The increased risk of sleep-related disorders in KD patients is an important finding that warrants further investigation. Sleep disturbances can significantly impact a child’s quality of life, cognitive function, and overall health. The higher incidence of movement disorders in KD patients is intriguing and may suggest subtle neurological sequelae of the disease. This finding underscores the importance of long-term neurological follow-up in KD survivors, even in the absence of overt neurological symptoms during the acute phase.

Study findings regarding neurodevelopmental disorders present a complex picture. Surprisingly, we observed a lower incidence of intellectual disability in individuals with KD compared to the controls. This unexpected finding requires careful interpretation and further investigation. In contrast, a Taiwan study reported an increased risk of intellectual disability in KD patients [19]. However, the subgroup analysis revealed that this risk was lower in those who received IVIG, suggesting that the immunomodulatory and anti-inflammatory effects of IVIG may play a protective role [32,33]. Similarly, a Canada cohort study found that the risk of intellectual disability increased ten years after KD diagnosis, indicating that long-term follow-up may yield different outcomes [20]. This suggests that the relationship between KD and intellectual disability may evolve over time and warrants further investigation. Another possible explanation is that KD patients receive more intensive medical care and follow-up, potentially leading to earlier detection and intervention for developmental issues, which could mitigate the risk of intellectual disability. The increased risk of tic disorder in KD patients is an important finding that adds to the growing evidence of neurological involvement in KD. Tic disorders have been associated with other inflammatory conditions, and this link with KD further supports the potential role of inflammation in the pathogenesis of tic disorders [34].

Interestingly, our study did not find significant associations between KD and psychotic disorders, mood disorders, cognitive disorders, and personality disorders. Likewise, among the neurodevelopmental disorders, we found no significant associations between KD and autism spectrum disorder and ADHD, which contrasts with findings from the Taiwan and Canada cohort studies. This selective pattern of neuropsychiatric risk suggests that KD may affect specific neural pathways or regions rather than cause widespread neuropsychiatric dysfunction.

Demographic factor analyses revealed that the risk of developing neuropsychiatric and neurodevelopmental disorders varied depending on specific disease characteristics, age at KD onset, sex, and residential area. Patients diagnosed with KD at ≥5 years had a lower risk of these disorders than those diagnosed at ≤1 year. This aligns with a Taiwan study linking early-onset KD (≤5 years) to higher cerebrovascular and neurological risks [5,35]. One possible explanation is that younger children experience heightened systemic inflammation during a critical period of neuroplasticity, potentially disrupting synaptic pruning and neural circuit maturation [36,37,38,39]. Male patients had a high incidence of neuropsychiatric and neurodevelopmental disorders, which may be related to the increased prevalence of KD complications in males [40]. Biological factors such as sex hormones like estrogen and testosterone modulate immune regulation and neurotransmitters, such as glutamate, dopamine, and GABA, contributing to differences in mental health outcomes [41,42,43]. Additionally, genetic factors may play a role, as the Y chromosome is implicated in dopamine system regulation, potentially increasing susceptibility in males [44]. Urban residency was associated with a greater risk of neuropsychiatric disorders. Previous studies have also reported that the urban environment is associated with an increased risk for mental illness [45,46]. This could be driven by environmental factors such as air pollution, chronic stress, and higher diagnostic awareness in urban healthcare settings. Further studies integrating immunological, neurodevelopmental, and environmental factors are needed to clarify the specific pathways underlying these associations.

The mechanisms underlying the association between KD and neuropsychiatric/neurodevelopmental disorders remain incompletely understood. However, growing evidence suggests that KD-induced systemic inflammation disrupts the blood–brain barrier (BBB), allowing cytokines and immune cells to enter the CNS, leading to neuronal damage and neurotransmitter alterations [5,47]. Elevated levels of inflammatory cytokines, such as IL-6 and TNF-α, have been detected in the CSF of KD patients, suggesting an ongoing inflammatory response within the CNS [7,13,48,49,50,51,52]. Chronic inflammation and vascular remodeling, which are well documented in KD-affected coronary arteries, may similarly affect the cerebral vasculature, contributing to long-term neuroinflammatory sequelae [35]. This inflammation may selectively affect vulnerable neurocircuits, contributing to the association between KD and specific psychiatric disorders. Additionally, studies linking mental health disorders with inflammatory pathways, as well as microbiome dysbiosis, provide further support for a shared pathological basis between KD and neuropsychiatric conditions [53,54,55]. Matrix metalloproteinases (MMPs), implicated in KD pathogenesis and psychiatric disorders, suggest potential molecular-level intersections [56,57,58,59]. Earlier studies documenting hypoperfusion in acute KD through SPECT imaging further reinforce the possibility of transient but significant cerebrovascular involvement during the disease’s acute phase [8].

Our findings suggest a link between KD and anxiety disorders, but we did not classify neuropsychiatric outcomes by KD subgroups, such as coronary artery involvement or systemic manifestations. Anxiety in KD patients may stem not only from molecular or inflammatory mechanisms but also from psychological stress related to complications, long-term health risks, or ongoing medical follow-up [60]. This highlights the multifactorial nature of the association rather than diminishes the role of biological mechanisms. Future studies should distinguish psychological and biological factor contributors to anxiety in KD patients through longitudinal or stratified analyses.

Despite these findings, our study has several limitations. First, the analysis was based solely on ICD-10 codes from administrative data, which may be prone to misclassification or underdiagnosis, particularly for neuropsychiatric and neurodevelopmental disorders that can have variable diagnostic criteria and healthcare access disparities. Second, we were unable to account for key confounding factors such as socioeconomic status, environmental exposures, and healthcare accessibility, all of which could have influenced the observed associations. Third, we did not include clinical parameters such as inflammatory markers, fever duration, or the severity of KD complications, which may provide additional insights into the mechanisms linking KD to neuropsychiatric disorders. While our study identifies a significant association between KD and neuropsychiatric/neurodevelopmental disorders, its observational nature prevents us from establishing a causal relationship. Future research with longer follow-up periods, biomarker analysis, and interventional trials is needed to clarify the causal relationship between KD and neuropsychiatric disorders.

## 5. Conclusions

This national cohort study demonstrates that KD is associated with an increased risk of specific neuropsychiatric disorders, including anxiety, sleep-related, movement, and tic disorders, while showing a lower incidence of intellectual disability. These findings highlight possible long-term neurological impacts of KD, possibly driven by systemic inflammation and immune dysregulation. From a clinical perspective, our results underscore the importance of long-term neurological monitoring and mental health surveillance in KD patients, even after the resolution of acute symptoms. Given the selective pattern of the neuropsychiatric risks observed, targeted follow-up strategies may help in the early identification and management of vulnerable subgroups. By advancing our understanding of the long-term neurological effects of KD, this study contributes to a growing body of research highlighting the interplay between systemic inflammation and brain health. Continued research will be crucial in refining risk stratification, optimizing follow-up strategies, and improving long-term outcomes for KD patients.

## Figures and Tables

**Table 1:** Baseline characteristics of the study subjects.

Variables	KD Cases (n = 41,806)	Controls (n = 163,829)	*p*-Value
Age			
Mean (years)	2.63 ± 1.84	2.64 ± 1.85	0.119
≤2 years	22,038 (52.71)	85,992 (52.49)	0.225
3–4 years	13,376 (32.00)	52,226 (31.88)	
≥5 years	6392 (15.29)	25,611 (15.63)	
Sex (female)	17,643 (42.20)	69,709 (42.55)	0.199
Urbanicity			0.025
Urban	28,054 (67.11)	108,991 (66.53)	
Non-urban	13,752 (32.89)	54,838 (33.47)	

Represented as mean ± SD or n (%). KD: Kawasaki disease.

**Table 2:** Association of neuropsychiatric disorders with Kawasaki disease.

	Kawasaki Disease		Univariate Analysis		Multivariate Analysis	
Case (n, %)	No	Yes	HR (95% CI)	*p*-Value	HR (95% CI)	*p*-Value
Psychotic disorder (−)	163,536 (95.82)	41,722 (99.80)	1.000		1.000	
Psychotic disorder (+)	293 (0.18)	84 (0.20)	1.096 (0.859–1.400)	0.459	1.097 (0.859–1.400)	0.459
Mood disorder (−)	161,639 (98.66)	41,218 (98.59)	1.000		1.000	
Mood disorder (+)	2190 (1.34)	588 (1.41)	1.068 (0.975–1.170)	0.156	1.074 (0.980–1.176)	0.126
Anxiety disorder (−)	160,403 (97.91)	40,835 (97.68)	1.000		1.000	
Anxiety disorder (+)	3426 (2.09)	971 (2.32)	1.124 (1.046–1.207)	0.001	1.124 (1.047–1.207)	0.001
Sleep-related disorder (−)	163,027 (99.51)	41,539 (99.36)	1.000		1.000	
Sleep-related disorder (+)	802 (0.49)	267 (0.64)	1.254 (1.091–1.441)	0.001	1.257 (1.094–1.444)	0.001
Cognitive disorder (−)	158,718 (96.88)	40,446 (96.75)	1.000		1.000	
Cognitive disorder (+)	5111 (3.12)	1360 (3.25)	1.046 (0.985–1.111)	0.140	1.049 (0.988–1.114)	0.118
Movement disorder (−)	163,309 (99.68)	41,636 (99.59)	1.000		1.000	
Movement disorder (+)	520 (0.32)	170 (0.41)	1.222 (1.026–1.454)	0.025	1.227 (1.030–1.461)	0.022
Personality disorder (−)	163,439 (96.76)	41,686 (99.71)	1.000		1.000	
Personality disorder (+)	390 (0.24)	120 (0.29)	1.219 (0.993–1.496)	0.058	1.215 (0.990–1.492)	0.063
Any disorder (−)	155,234 (94.75)	39,400 (94.24)	1.000		1.000	
Any disorder (+)	8595 (5.25)	2406 (5.76)	1.101 (1.053–1.152)	<0.001	1.102 (1.053–1.153)	<0.001

**Table 3:** Association of neurodevelopmental disorders with Kawasaki disease.

	Kawasaki Disease		Univariate Analysis		Multivariate Analysis	
Case (n, %)	No	Yes	HR (95% CI)	*p*-Value	HR (95% CI)	*p*-Value
Intellectual disorder (−)	162,817 (99.38)	41,612 (99.54)	1.000		1.000	
Intellectual disorder (+)	1012 (0.62)	194 (0.46)	0.747 (0.640–0.871)	<0.001	0.747 (0.641–0.871)	<0.001
Communication disorder (−)	163,051 (99.53)	41,628 (99.57)	1.000		1.000	
Communication disorder (+)	778 (0.47)	178 (0.43)	0.897 (0.762–1.056)	0.192	0.894 (0.760–1.052)	0.179
Specific learning disorder (−)	163,708 (99.93)	41,785 (99.95)	1.000		1.000	
Specific learning disorder (+)	121 (0.07)	21 (0.05)	0.643 (0.400–1.033)	0.068	0.641 (0.399–1.030)	0.066
Autism spectrum disorder (−)	163,226 (99.63)	41,670 (99.67)	1.000		1.000	
Autism spectrum disorder (+)	603 (0.37)	136 (0.33)	0.876 (0.727–1.056)	0.165	0.872 (0.724–1.051)	0.150
ADHD (−)	160,512 (97.98)	40,951 (97.95)	1.000		1.000	
ADHD (+)	3317 (2.02)	855 (2.05)	1.021 (0.947–1.101)	0.580	1.017 (0.943–1.096)	0.663
Tic disorder (−)	162,608 (99.25)	41,451 (99.15)	1.000		1.000	
Tic disorder (+)	1221 (0.75)	355 (0.85)	1.154 (1.025–1.298)	0.018	1.148 (1.020–1.292)	0.022
Any disorder (−)	157,926 (96.40)	40,262 (96.31)	1.000		1.000	
Any disorder (+)	5903 (3.60)	1544 (3.69)	1.029 (0.973–1.088)	0.322	1.027 (0.971–1.086)	0.361

HR: hazard ratio; ADHD: attention deficit hyperactivity disorder.

## Data Availability

The data that support the findings of this study were obtained from the National Health Insurance Service (NHIS) of Korea under strict confidentiality agreements. Due to the privacy regulations and data sharing policies of the NHIS, these data cannot be shared publicly. Researchers interested in accessing the data can request access directly from the NHIS (https://nhiss.nhis.or.kr).

## Supplementary Materials

The following supporting information can be downloaded at https://www.mdpi.com/article/10.3390/medicina61040604/s1, Table S1: Diagnostic code of neuropsychiatric and neurodevelopmental disorders; Table S2: Comparison of the number of prevalent cases in neuropsychiatric disorders; Table S3: Comparison of the number of prevalent cases in neurodevelopmental disorders.

## Abbreviations

The following abbreviations are used in this manuscript:

KD	Kawasaki Disease
ADHD	Attention Deficit Hyperactivity Disorder
HR	Hazard Ratio
OR	Odds Ratio
SPECT	Single-Photon Emission Computed Tomography
NHIS	National Health Insurance Service
