# Supplementary material for: Linking Kawasaki Disease to Mental Health: A Nationwide Study on Long-Term Neurological Risks
Source: Medicina (Kaunas). 2025 Mar 26;61(4):604. doi: 10.3390/medicina61040604 (PMC12028643; doi:10.3390/medicina61040604)
Supplement: Supplementary file 1 [file medicina-61-00604-s001.zip › medicina-3540313-supplementary.pdf]

Table S1. Diagnostic code of neuropsychiatric and neurodevelopmental disorders

**Neuropsychiatric disorders**	**Diagnostic code**
Psychotic disorder	F20, F21, F22, F23, F24, F25, F28, F29, R410, R44
Mood disorder	F30, F31, F32, F33, F34, F38, F39
Anxiety disorder	F40, F41, F42, F43, F44, F45, F48, R451
Sleep-related disorder	F51, G47
Cognitive disorder	R411, R412, R413, F90, F98
Movement disorder	G20, G21, G24, G25
Personality disorder	F60, F61, F62, F63, F64, F65, F66, F68, F69, F94
**Neurodevelopmental disorders**	**Diagnostic code**
Intellectual Disability	F70–F79
Communication disorders	F800, F801, F802, F804, F809
Specific Learning Disorder	F810, F811, F812, F813, F819
Autism Spectrum Disorder	F840, F841, F845, F849
ADHD	F90.0–F90.9
Tic Disorders	F950, F951, F952, F959

ADHD: Attention Deficit Hyperactivity Disorder.

Table S2. Comparison of the number of prevalent cases in neuropsychiatric disorder.

Case (n, %)	Kawasaki disease		Univariate analysis		Multivariate analysis	
	No	Yes	OR (95% CI)	*p*	OR (95% CI)	*p*
Psychotic disorder (-)	163,517 (95.81)	41,721 (99.80)	1.000		1.000	
Psychotic disorder (+)	312 (0.19)	85 (0.20)	1.068 (0.840–1.357)	0.592	1.064 (0.837–1.352)	0.614
Mood disorder (-)	161,503 (98.58)	41,171 (98.48)	1.000		1.000	
Mood disorder (+)	2,326 (1.42)	635 (1.52)	1.071 (0.980–1.170)	0.129	1.068 (0.978–1.167)	0.142
Anxiety disorder (-)	160,153 (97.76)	40,764 (97.51)	1.000		1.000	
Anxiety disorder (+)	3,676 (2.24)	1,042 (2.49)	1.114 (1.039–1.194)	0.002	1.112 (1.037–1.192)	0.003
Sleep-related disorder (-)	162,967 (99.47)	41,514 (99.30)	1.000		1.000	
Sleep-related disorder (+)	862 (0.53)	292 (0.70)	1.330 (1.164–1.520)	<0.001	1.328 (1.163–1.518)	<0.001
Cognitive disorder (-)	157,944 (96.41)	40,239 (96.25)	1.000		1.000	
Cognitive disorder (+)	5,885 (3.59)	1,567 (3.75)	1.045 (0.987–1.106)	0.127	1.041 (0.983–1.102)	0.168
Movement disorder (-)	163,284 (99.67)	41,622 (99.56)	1.000		1.000	
Movement disorder (+)	545 (0.33)	184 (0.44)	1.325 (1.121–1.566)	0.001	1.324 (1.120–1.565)	0.001
Personality disorder (-)	163,415 (96.75)	41,681 (99.70)	1.000		1.000	
Personality disorder (+)	414 (0.25)	125 (0.30)	1.184 (0.969–1.446)	0.099	1.179 (0.965–1.441)	0.107
Any disorder (-)	153,810 (93.88)	38,997 (93.28)	1.000		1.000	
Any disorder (+)	10,019 (6.12)	2,809 (6.72)	1.106 (1.059–1.155)	<0.001	1.104 (1.057–1.153)	<0.001

OR: Odds Ratio.

Table S3. Comparison of the number of prevalent cases in neurodevelopmental disorder.

Case (n, %)	Kawasaki disease		Univariate analysis		Multivariate analysis	
	**No**	**Yes**	**OR (95% CI)**	***p***	**OR (95% CI)**	***p***
Intellectual disorder (-)	162,548 (99.22)	41,553 (99.39)	1.000		1.000	
Intellectual disorder (+)	1,281 (0.78)	253 (0.61)	0.773 (0.675–0.885)	<0.001	0.771 (0.674–0.883)	<0.001
Communication disorder (-)	162,853 (99.40)	41,580 (99.46)	1.000		1.000	
Communication disorder (+)	976 (0.60)	226 (0.54)	0.907 (0.784–1.049)	0.187	0.904 (0.782–1.045)	0.171
Specific learning disorder (-)	163,704 (99.92)	41,783 (99.94)	1.000		1.000	
Specific learning disorder (+)	125 (0.08)	23 (0.06)	0.721 (0.462–1.125)	0.150	0.718 (0.460–1.121)	0.145
Autism spectrum disorder (-)	162,928 (99.45)	41,595 (99.50)	1.000		1.000	
Autism spectrum disorder (+)	901 (0.55)	211 (0.50)	0.917 (0.789–1.066)	0.260	0.913 (0.786–1.061)	0.236
ADHD (-)	160,090 (97.72)	40,833 (97.67)	1.000		1.000	
ADHD (+)	3,739 (2.28)	973 (2.33)	1.020 (0.950–1.096)	0.580	1.015 (0.945–1.091)	0.681
Tic disorder (-)	162,511 (99.20)	41,411 (99.06)	1.000		1.000	
Tic disorder (+)	1,318 (0.80)	395 (0.94)	1.176 (1.051–1.317)	0.005	1.172 (1.046–1.312)	0.006
Any disorder (-)	156,192 (95.34)	39,826 (95.26)	1.000		1.000	
Any disorder (+)	7,637 (4.66)	1,980 (4.74)	1.017 (0.967–1.070)	0.517	1.013 (0.962–1.065)	0.632

OR: Odds Ratio; ADHD: Attention Deficit Hyperactivity Disorder.
